# Osseous differentiation of human fat tissue grafts: From tissue engineering to tissue differentiation

**DOI:** 10.1038/srep39712

**Published:** 2017-01-05

**Authors:** Maryna Bondarava, Chiara Cattaneo, Bin Ren, Wolfgang E. Thasler, Volkmar Jansson, Peter E. Müller, Oliver B. Betz

**Affiliations:** 1 University Hospital of Munich (LMU), Campus Grosshadern, Department of Orthopedic Surgery, Physical Medicine and Rehabilitation, Munich, DE, Germany; 2 University Hospital of Munich (LMU), Biobank under the administration of the Human Tissue and Cell Research (HTCR) Foundation, Department of General, Visceral, Transplantation, Vascular and Thoracic Surgery, Munich, DE, Germany.

## Abstract

Conventional bone tissue engineering approaches require isolation and *in vitro* propagation of autologous cells, followed by seeding on a variety of scaffolds. Those protracted procedures impede the clinical applications. Here we report the transdifferentiation of human fat tissue fragments retrieved from subcutaneous fat into tissue with bone characteristics *in vitro* without prior cell isolation and propagation. 3D collagen-I cultures of human fat tissue were cultivated either in growth medium or in osteogenic medium (OM) with or without addition of Bone Morphogenetic Proteins (BMPs) BMP-2, BMP-7 or BMP-9. Ca^2+^ depositions were observed after two weeks of osteogenic induction which visibly increased when either type of BMP was added. mRNA levels of alkaline phosphatase (ALP) and osteocalcin (OCN) increased when cultured in OM alone but addition of BMP-2, BMP-7 or BMP-9 caused significantly higher expression levels of ALP and OCN. Immunofluorescent staining for OCN, osteopontin and sclerostin supported the observed real-time-PCR data. BMP-9 was the most effective osteogenic inducer in this system. Our findings reveal that tissue regeneration can be remarkably simplified by omitting prior cell isolation and propagation, therefore removing significant obstacles on the way to clinical applications of much needed regeneration treatments.

A growing aging population with an increased risk of bone fractures due to falls^1^, unfortunately often combined with impaired bone healing and even higher fracture risk due to osteoporosis^2^ and diabetes^3^,^4^, and, in addition, to the currently inevitable loosening of prosthetic implants over time^5^, urgently requires adequate bone regeneration strategies. The situation is exacerbated by a rising number of devastating traumatic war injuries for which often no other treatment option than amputation exists due to the extensive loss of bone and soft tissue^6^. Current treatment options are associated with high morbidity^7^,^8^,^9^ or deficient efficacy^10^. Mesenchymal stem cells (MSCs) in combination with various scaffolds are under intensive investigation and show promising achievements^11^,^12^,^13^,^14^. However, the involved procedure of cell isolation or separation is costly, leading to an estimated market worth 6.3 billion USD by 2020^15^. Current tissue engineering strategies also involve subsequent *in vitro* propagation of the prior isolated or separated cells. These procedures add further substantial costs which is reflected in an estimated market worth 14.8 billion USD by 2019^16^. Extended cultivation time also holds a concerning higher risk of contamination and unwanted effects due to prolonged exposure to the cell culture media^17^. The cell propagation is currently done in external GMP-Facilities, which again adds costs, time and risks to the cell product. To avoid the issues afflicted with the use of an external GMP-Facility, the idea of a “GMP in a box”, in form of a fully automated benchtop culture system within the primary-care facility of the patient seems very promising^18^. Not only would the use of tissue grafts instead of isolated and propagated cells clearly simplify such a system, therefore accelerating the availability, but because even when performed in the operating room and used autologously, cell separation is considered “more than minimally manipulated” by the FDA^15^, requiring a more rigorous approval process. Tissue grafts could therefore remove another obstacle on the road to a clinical application. Lastly, fat tissue grafts have the potential to further the demand by surgeons for a same day, *ex-vivo* therapy^19^. For all those reasons above, it becomes obvious that technologies without the requirement of cell isolation and propagation would increase the chances to meet the need of the increasing number of patients for bone regeneration. Preclinical studies, showing that implantation of fat or muscle tissue fragments transduced with an adenoviral BMP-2 vector induces structural and functional healing of large segmental bone defects, were recently reported^20^,^21^,^22^,^23^. However, it remained unclear, whether the BMP-2 transduced tissue graft itself can undergo transdifferentiation into bone or if the graft rather serves as a delivery system for growth factors which stimulate and attract stem cells of the surrounding tissue^21^. If the latter would be the case, it would be advised to focus rather on drug delivery optimisation than cell therapies. Therefore, the aim of the present study was to investigate whether human fat tissue containing inhomogeneous cell populations is capable of transdifferentiation into tissue with bone characteristics. Adipose tissue harvest is associated with minimal donor site morbidity and it represents an especially appealing source of progenitor cells that can be used for the repair of bone^21^,^24^. A new culture system, introduced by Sonoda *et al*.^25^, represents adipose tissue fragments embedded in a three-dimensional collagen gel. This culture method was preferred in the present study not only because of the ability to easily entrap buoyant adipose tissue fragments, but also due to the support of a three-dimensional tissue structure mimicking an *in vivo* situation more closely and providing favourable conditions for cell differentiation. Bone morphogenetic proteins (BMPs) are known as bone forming growth factors. BMP-2 and BMP-7 have been shown to induce osteogenesis *in vivo*^26^,^27^,^28^,^29^,^30^,^31^,^32^,^33^,^34^, and both are available for clinical use^35^,^36^,^37^. Another promising growth factor inducing formation of bone is BMP-9. The superior potential of BMP-9 to induce osteogenic differentiation of MSCs was demonstrated both *in vivo* and *in vitro*^38^,^39^,^40^,^41^,^42^,^43^,^44^,^45^,^46^. However, no studies comparing the influence of BMP-2, BMP-7 and BMP-9 on human fat tissue fragments are available so far.

In this study, we used a modified, three-dimensional collagen I gel system to culture human subcutaneous fat tissue fragments *in vitro*. We evaluated whether cultivation with osteogenic supplements leads to tissue calcification and expression of osteoblast specific protein markers. Additionally, the potential of three recombinant growth factors BMP-2, BMP-7 and BMP-9 to induce osteogenic transdifferentiation of fat tissue was explored and compared.

## Results

### 3-Dimentional fat tissue culture system

**Figure 1:**
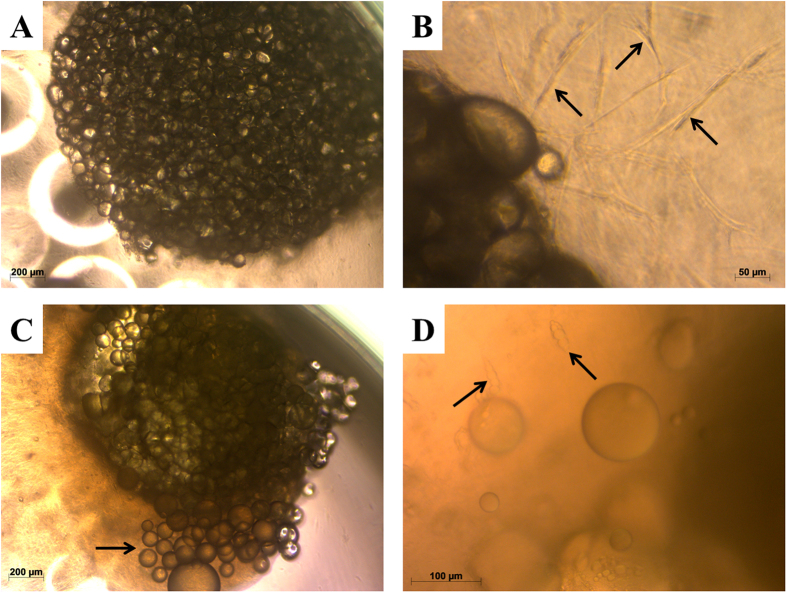
Phase contrast images of collagen-I fat cultures. (**A**) Freshly prepared culture. (**B**) Migration of spindle-shaped cells (arrows) out of the fat tissue in the collagen-I gel after 1 week of culture. (**C**) Migration of adipocytes (arrow) out of the fat tissue after 2 weeks of culture. (**D**) Spindle-shaped cells containing fat droplets (arrows) migrating into the collagen-I gel after 3 weeks of culture.

After being embedded in collagen I gel, adipose tissue fragments appeared under the phase contrast microscope as mature adipocytes attached to each other (Fig. 1A). After 5–7 days in culture, spindle-shaped cells were observed at the peripheral zones of the fragments migrating into the collagen I gel (Fig. 1B). Furthermore, after the second week of culture, some adipocytes started to dissociate from the tissue conglomerate. This could be observed exceedingly in samples cultured in normal growth medium (MN) (Fig. 1C). In addition to adipocytes and spindle-shaped cells, some preadipocyte-like cells with small fat droplets were observed in the late stages of the culture period of 4 weeks (Fig. 1D). Collagen-I gel was continuously shrinking during the whole period of incubation and losing its transparency under the osteogenic conditions. The migrating cells were spread throughout the collagen gel, partly covering the bottom surface of the 96-well plates.

### Cell viability and proliferation

**Figure 2:**
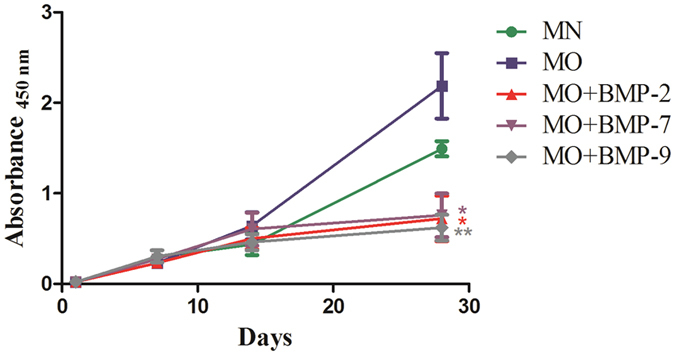
WST-1 measurement of fat tissue cultures under different differentiation culture conditions. Values given represent means ± SE, n = 6–7. The level of significance was set as * for p < 0.05, ** for p < 0.01 and *** for p < 0.001.

Cell viability and proliferation in the fat fragments was not only maintained for four weeks, but increased progressively over time in all experimental groups (Fig. 2). The highest WST-1 absorbance values, as an indicator of cell viability and proliferation, were observed in the osteogenic medium (MO) group: 0.23 ± 0.037 after 1 week, 0.64 ± 0.15 after 2 weeks, and 2.19 ± 0.36 after 4 weeks of incubation. Less increase in absorbance was observed in cultures treated with normal media (MN): 0.29 ± 0.06 after 1 week, 0.44 ± 0.12 after 2 weeks, and 1.49 ± 0.08 after 4 weeks of incubation. However, no significant difference was detected between the MO and MN groups. In contrast, significant lower absorbance values in comparison to MN were detected in all groups treated with BMPs after 4 weeks of incubation: 0.23 ± 0.04; 0.50 ± 0.12; 0.72 ± 0.25 (p ≤ 0.05) after 1, 2 and 4 weeks respectively in the MO-BMP-2 group; 0.27 ± 0.04; 0.61 ± 0.18; 0.76 ± 0.24 (p ≤ 0.05) after 1, 2 and 4 weeks respectively in the MO-BMP-7 group; 0.30 ± 0.07; 0.46 ± 0.09; 0.62 ± 0.14 (p ≤ 0.01) after 1, 2 and 4 weeks respectively in the MO-BMP-9 group, indicating a significant lower proliferation rate in the BMP receiving groups. No significant difference between the BMP-groups was observed.

### Histology

**Figure 3:**
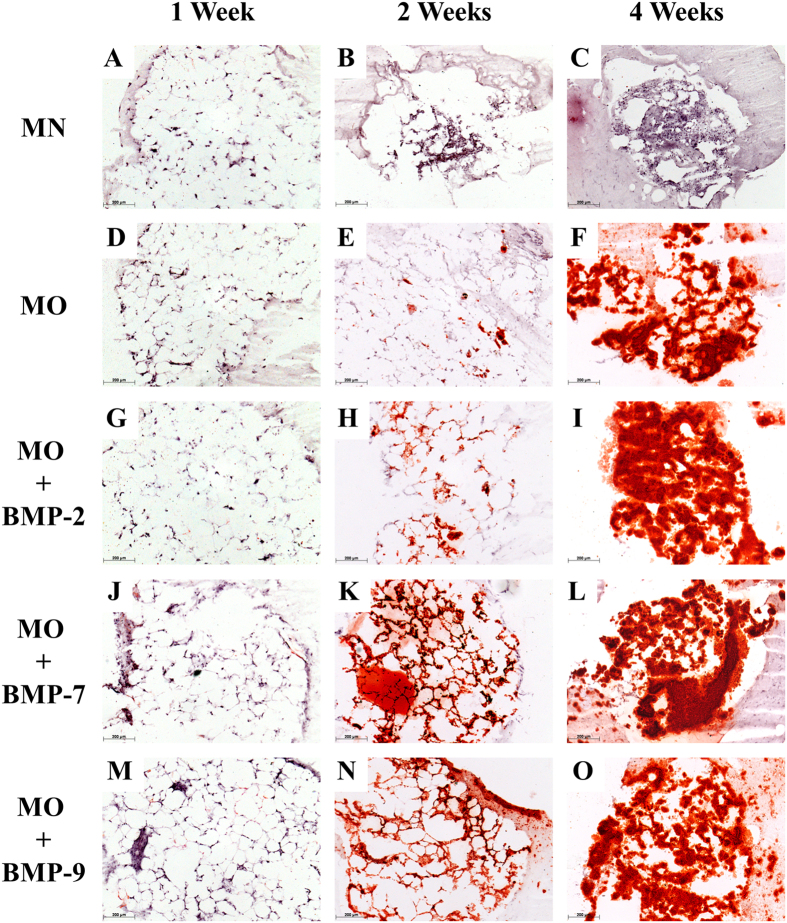
Histological sections of fat cultures after 1, 2 or 4 weeks under various differentiation conditions. Sections were stained with Alizarin red S: 1 week: (**A**) MN, (**D**) MO, (**G**) MO + BMP-2, (**J**) MO + BMP-7, (**M**) MO + BMP-9; 2 weeks: (**B**) MN, (**E**) MO, (**H**) MO + BMP-2, (**K**) MO + BMP-7, (**N**) MO + BMP-9; and 4 weeks of incubation: (**C**) MN, (**F**) MO, (**I**) MO + BMP-2, (**L**) MO + BMP-7, (**O**) MO + BMP-9. Scale bar = 200 μm.

**Figure 4:**
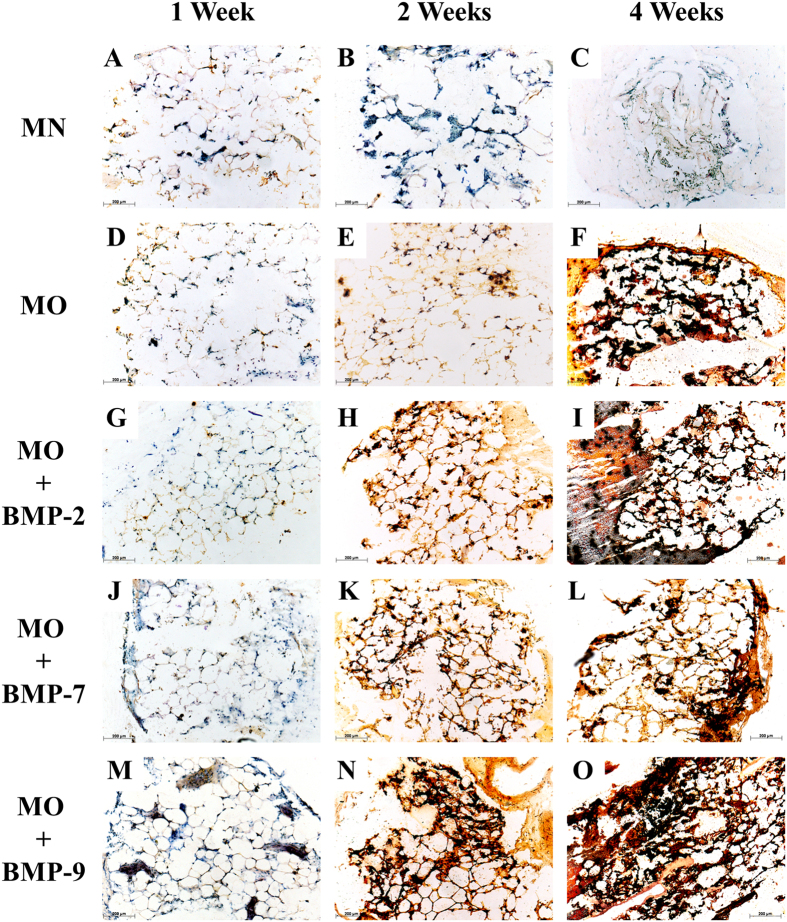
Histological sections of fat cultures after 1, 2 or 4 weeks under various differentiation conditions. Sections were stained with von Kossa: 1 week: (**A**) MN, (**D**) MO, (**G**) MO + BMP-2, (**J**) MO + BMP-7, (**M**) MO + BMP-9; 2 weeks: (**B**) MN, (**E**) MO, (**H**) MO + BMP-2, (**K**) MO + BMP-7, (**N**) MO + BMP-9; and 4 weeks of incubation: (**C**) MN, (**F**) MO, (**I**) MO + BMP-2, (**L**) MO + BMP-7, (**O**) MO + BMP-9. Scale bar = 200 μm.

After one week of incubation, none of the five groups displayed calcium depositions. (Figs 3A,D,G,J,M and 4A,D,G,J,M). After the second week of incubation, the samples treated with MN showed a higher number of cell nuclei located between the adipocytes than compared to cultures under osteogenic conditions. This suggests a higher proliferation of stromal cells in the MN group. Samples treated with MO displayed some regions of calcification located in the stromal cell fraction between adipocytes. The fat grafts of the BMP-2, -7 and -9 groups displayed a remarkable increase in extracellular calcification, as confirmed by alizarin red S as well as by von Kossa staining (Figs 3H,I,K,L,N,O and 4H,I,K,L,N,O). After the fourth week of incubation the proliferation of the stromal cells in the MN group increased even further replacing adipocytes almost completely. The samples treated with MO and MO + BMPs demonstrated a strong overall tissue calcification, which included the adjacent collagen I gel. There was no significant difference in the calcification rate observed between MO and MO + BMP-2, -7 or -9 experimental groups after the 4^th^ week of incubation.

### Real-time quantitative PCR (RT-PCR)

**Figure 5:**
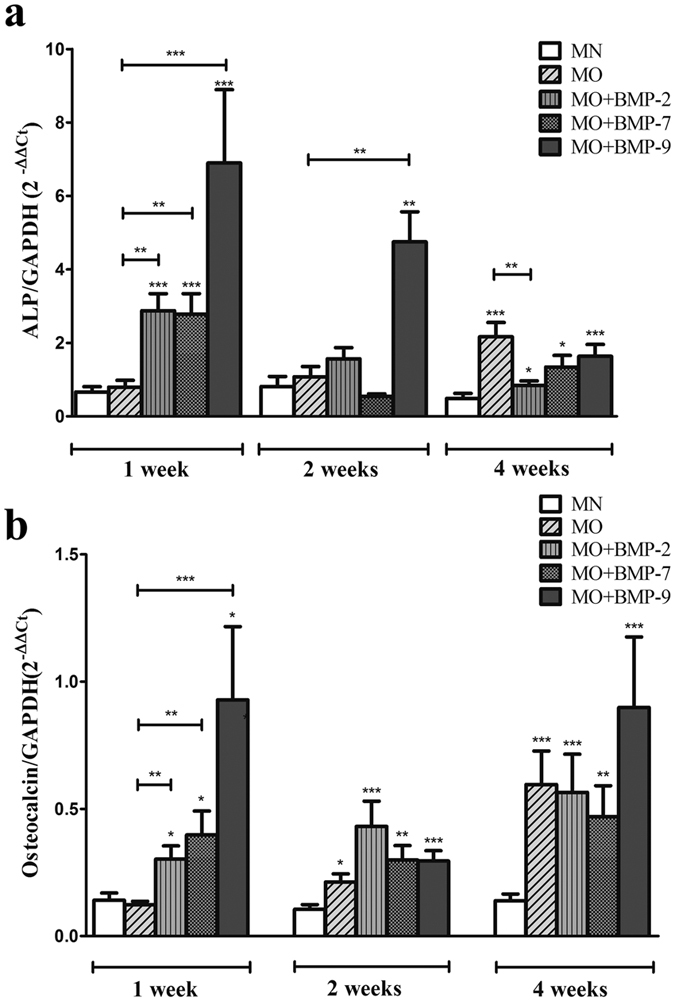
Quantitative RT-PCR analysis of 3D fat cultures incubated under different differentiation conditions. Expression of mRNA for ALP (**a**) and OCN (**b**) normalized to mRNA levels for GAPDH. Values given represent means ± SE, n = 10–11. The level of significance was set as * for p < 0.05, ** for p < 0.01 and ***for p < 0.001.

Osteogenic differentiation of the 3D cultures was confirmed by quantitative real-time PCR measuring the expression of the early bone marker ALP and the later bone marker OCN mRNA. The expression of these markers in native fat tissue of each patient served as a baseline (calibrator). The mRNA expression of the early bone marker ALP after one week of culture was significantly (p ≤ 0.001) higher in samples treated with MO plus BMP-2 (2.87 ± 0.46), BMP-7 (2.78 ± 0.56) and BMP-9 (6.90 ± 2.00) compared to samples treated with MN (0.66 ± 0.15) or MO (0.80 ± 0.19, p ≤ 0.01 or p ≤ 0.001). The ALP-expression for MO plus BMP-9 was significantly higher than for all other groups (Fig. 5a). After the second week of culture the level of ALP in the MO + BMP-9 group was still significantly higher (4.75 ± 0.82, p ≤ 0.01) than in the other groups: MN (0.81 ± 0.27), MO (1.08 ± 0.28), MO + BMP-2 (1.56 ± 0.31), MO + BMP-7 (0.55 ± 0.07). However, after the fourth week of incubation the highest level of ALP expression was observed in the MO group (2.16 ± 0.39, p ≤ 0.001). The groups MO + BMP-2, MO + BMP-7 and MO + BMP-9 showed significantly higher ALP expression values (0.84 ± 0.12; 1.35 ± 0.31; 1.64 ± 0.32 respectively, p ≤ 0.05 or p ≤ 0.001) compared to the MN group (0.48 ± 0.14) as well (Fig. 5a).

OCN mRNA expression after the first week of culture was significantly higher in the samples treated with MO containing BMP-2 (0.30 ± 0.05), BMP-7 (0.40 ± 0.09) or BMP-9 (0.93 ± 0.29) than in samples treated with MN (0.14 ± 0.03, p ≤ 0.05) or MO (0.12 ± 0.02, p ≤ 0.01 or p ≤ 0.001) (Fig. 5b). After the second week of culture the level of OCN expression was significantly higher in the samples treated with MO (0.21 ± 0.03, p ≤ 0.05), MO + BMP-2 (0.43 ± 0.10, p ≤ 0.001), MO + BMP-7 (0.30 ± 0.06, p ≤ 0.01) and MO + BMP-9 (0.29 ± 0.04, p ≤ 0.001) compared to the samples treated with MN (0.11 ± 0.02). After the fourth week of incubation the OCN mRNA was significantly overexpressed in the groups MO, MO + BMP-2, MO + BMP-7 and MO + BMP-9 (0.60 ± 0.03, p ≤ 0.001; 0.56 ± 0.13, p ≤ 0.001; 0.47 ± 0.12, p ≤ 0.01 and 0.90 ± 0.28, p ≤ 0.001 respectively) compared to to the MN group (0.14 ± 0.03) (Fig. 5b).

### Immunofluorescence

**Figure 6:**
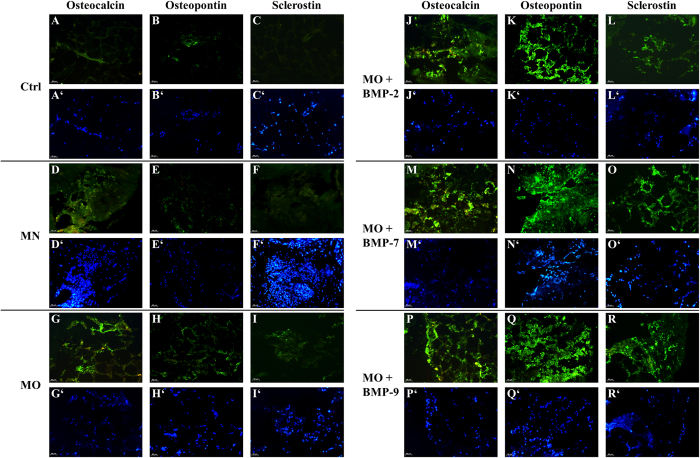
Histological sections of fat cultures after 4 weeks under various differentiation conditions. Immunofluorescence for OCN, OPN and Scl is shown in green. Control (Ctrl) samples (**A**–**C**) represent the untreated fat fragments. Samples treated with MN are shown in the images (**D**–**F**); MO at (**G**–**I**); MO + BMP-2 in (**J**–**L**); MO + BMP-7 in (**M**–O); MO + BMP-9 in (**P**–**R**). Nuclei of cells (blue) are shown in the corresponding images (**A’**–**R’**). Scale bar = 50 μm.

Immunofluorescent staining of native fat tissue fragments for the bone markers osteocalcin (OCN) and osteopontin (OPN) showed that these proteins were endogenously expressed at very low levels in the adjacent blood vessels and in some niches of the stromal cells within fat tissue, but not in the adipocytes (Fig. 6A,B). In contrast, sclerostin could not be detected in native fat samples (Fig. 6C). After 4 weeks of culture in MN, the bone markers OCN and OPN could still be detected intracellular, although at minimal levels, mostly localized in the cell nuclei (Fig. 6D,E). Expression of sclerostin was not observed in MN cultures (Fig. 6C,F). Samples treated with MO displayed a low fluorescence signal for OCN and OPN (Fig. 6G,H) and a positive signal for sclerostin (Fig. 6I). All the samples treated with MO and supplemented with BMPs (BMP-2, BMP-7, BMP-9) showed strong fluorescence signals for OCN, OPN and sclerostin (Fig. 6J–R). There was no apparent difference observed concerning the expression of OCN and OPN between the three different BMPs. Sclerostin expression appeared to be higher in samples treated with MO + BMP-7 and MO + BMP-9 compared to MO + BMP-2 (Fig. 6L,O,R). Furthermore, fluorescence signals from OCN, OPN and sclerostin in samples treated with MO + BMPs were observed not only intracellular, but also extracellular and to some extend in the surrounding collagen I matrix.

## Discussion

In this study we employed a 3D fat tissue culture system established by Sonoda *et al*.^25^ with minor adaptations. Despite our adaptations, most of the vitality and histological data were in line with the results found by Sonoda *et al*.^25^. The fat tissue cultures in collagen I gel did not only retain their viability but rather displayed increasing vitality parameters over 4 weeks. The migration and proliferation of spindle-shaped cells were observed at the periphery of the fat tissue fragments. Cells of the same phenotype were shown by Sonoda *et al*. (Fig. 1B) to express CD 44 and CD 105 – the surface markers of mesenchymal stem cells^25^. Capable of migration and proliferation, these cells might contribute to the osteogenic differentiation described in the present study. The dissociation of adipocytes at the late phase of the tissue culture was not shown before^25^. A possible reason could be the human origin of the tissue as opposed to the rat origin used by Sonoda and the difference in culture conditions. Only very few spindle-shaped cells containing lipid droplets (Fig. 1D) were observed in the cultures, making it highly unlikely, that these cells are exclusively responsible for the osteogenic differentiation of the fat grafts. This observation rather suggests the presence of preadipocytes or dedifferentiated adipocytes within the fat tissue. Moreover, the vitality of the 3D cultures was continuously increasing during the first two weeks of culture with no significant difference between the experimental groups (Fig. 2). However, after 4 weeks of incubation the vitality of groups containing recombinant BMPs (BMP-2, -7 and -9) was significantly lower than in MN and MO groups. Addition of osteogenic growth factors to the cultures caused an accelerated differentiation rate but also a decrease of the proliferation rate. This was to be expected as cell differentiation has long been recognised as an opposing process to cell proliferation^47^.

Accumulation of calcium under osteogenic conditions was observed mostly among mature adipocytes at the sites of stromal cell localisation, or in the niches of progenitor cells (Figs 3 and 4). Presumably, only these cells produce a sufficient number of progenitors that can respond to osteogenic stimuli and differentiate into the respective lineage. Moreover, calcification in samples containing BMPs was observed in the collagen I gel adjacent to the fat tissues, suggesting the contribution of migrated cells to the overall calcification (Figs 3L,O and 4I).

Calcium accumulation during osteogenic differentiation was already described in several studies on adipose-derived stem cells cultured in monolayer^24^,^48^ or in 3D cultures^49^,^50^. To the best of our knowledge, this reported study demonstrates for the first time, osteogenic differentiation, including calcification of 3D fat tissue cultures of human origin *in vitro*. Although, Betz *et al*.^20^ have demonstrated that BMP-2 gene-activated fat tissue is an osteoregenerative material that has the ability to repair critical-sized bone defects in rats^21^, it was still unclear, whether the gene-activated tissues expressing BMP-2 transform into bone or solely serve as a delivery system for BMP-2 stimulating and attracting stem cells of the surrounding tissue. The presented data substantiates our hypothesis that fat tissue has the potential for the transition into bone tissue.

The RT-PCR data reveals increased mRNA expression of the bone markers ALP and OCN under osteogenic culture conditions. Moreover, the addition of BMP-2, -7 and -9 seems to induce osteogenesis in the fat tissue earlier and more intensively than osteogenic medium alone. These data were supported by the immunofluorescent staining for OCN, OPN and Scl. Furthermore, Scl appears to be the most specific osteogenic marker in this system, as it was only detectable after osteogenic stimulation but not in the native or in MN cultured tissue. The presented data support the hypothesis, that osteogenesis can be induced in human fat tissue without isolation of progenitor cells. The presence of mesenchymal stem cells and their osteogenic potential were confirmed for different types of adipose tissue by numerous studies^48^,^50^,^51^. However, no data was reported so far on fat tissue transdifferentiation *in vitro*. Limited *in vivo* data reporting transdifferentiation between white and brown types of fat cells^52^ as well as fat-epithelial cell differentiation^53^,^54^ was reported. Moreover, Gao *et al*.^55^ described recent insights in studying transdifferentiation of bone and fat related to bone metabolism. Subsequent research revealed the capacity of subcutaneous preadipocytes to differentiate into osteoblasts^56^. Other studies reported, that mature lipid-filled adipocytes transdifferentiated into mature osteoblasts through the preadipocytic stage with increased cell proliferation^57^,^58^. Also, it was discovered that single adipocytes are capable to dedifferentiate into cells with fibroblast-like morphology and subsequently turn into osteoblasts or adipocytes under respective stimulation^58^,^59^.

The osteogenic BMPs hold great promise for regenerative medicine^60^. Our results have demonstrated that BMP-2, -7 and -9 represent osteogenic activities and enhance osteogenic differentiation of human fat tissue *in vitro* at the mRNA and protein level. Moreover, BMP-9 seems to be a more potent osteogenic inducer for cells within fat tissue than BMP-2 or -7. This observation is in line with the previous studies conducting comprehensive analysis of different types of human BMPs^39^,^41^,^61^,^62^. By using recombinant adenoviruses in order to facilitate the expression of 14 different BMPs, Luu *et al*., 2006 have demonstrated that, besides BMP-2 and BMP-7, BMP-6 and BMP-9 show the highest osteogenic activity *in vitro* as well as *in vivo*^39^. Li *et al*., 2003 have reported that BMP-9 displays the greatest osteogenic potential *in vitro* and in Sprague-Dawley rats^62^. Furthermore, Kang *et al*.^41^ suggested that high osteogenic activity of BMP-9 may be explained by transduction of a distinct osteogenic signalling pathway that is completely different from that of BMP-2, BMP-6 and BMP-7^41^. One reason for the osteogenic potency of BMP-9 is most likely the fact, that it cannot be inhibited by noggin, an effective inhibitor of BMP-2 and -7^63^,^64^. This would also explain why BMP-9 is involved in traumatic, heterotopic ossification where small amounts of BMP-9 can induce ossification of soft tissue in humans^65^.

In conclusion, we have demonstrated for the first time that human fat tissue is able to undergo osteogenic transdifferentiation under 3D culture conditions *in vitro,* although it remains unclear which compartments of the heterogenic cell population within the fat tissue are responsible for the differentiation process. We furthermore evaluated the osteogenic potential of human recombinant BMP-2, -7 and -9 in this system. Here, BMP-9 was identified to be the most potent osteogenic inducer. Our results suggest that human subcutaneous fat tissue may represent a regenerative material, which could be applied without cell isolation in expedited bone regeneration strategies^21^,^23^. Future investigations should be directed toward understanding the cellular and molecular mechanisms of fat tissue transdifferentiation *in vitro* and *in vivo* and also toward the role of BMPs in this context with a potential focus on BMP-9.

At the clinical research front, the combinations of different BMPs and other growth factors should be evaluated for synergic osteogenic activity. In addition, alternative and safe BMP- delivery approaches need to be developed to enable efficient, safe and cost effective therapies for bone regenerative medicine.

## Methods

### Tissue Harvest and 3-Dimentional Culture System

The tissues and data used in this study were provided by the Biobank (http://www.klinikum.uni-muenchen.de/Chirurgische-Klinik-und-Poliklinik-Grosshadern/de/0800-gewebebank/index.html) located in the Hospital of the University of Munich, which operates in accordance with the European Union-compliant ethical and legal framework of the Human Tissue and Cell Research (HTCR) Foundation (http://www.htcr.org). The process of tissue collection included obtaining written informed consent from all tissue donors. The experimental procedures were performed within the framework of the HTCR. This framework has also been approved by the ethics commission of the Faculty of Medicine in the University of Munich and the Bavarian State Medical Association^66^.

The subcutaneous fat tissue was obtained from 5 patients of different sex (age from 31–80, BMI 29–52). After rinsing with PBS containing 120 IU/ml penicillin/streptomycin and 0,375 μg/ml amphotericin B (Biochrom, Germany), the adipose tissue was punched with a biopsy punch to create fragments of approximately 2 mm in diameter. Each fragment was then embedded in 50 μl type I collagen gel solution (3D Collagen Culture Kit, Merck Millipore, Germany), which was prepared according to the manufacturer’s protocol, and placed in a 96-well tissue culture plate. After polymerization of the collagen, the cultures were covered either with normal, maintaining media (MN) (Ham’s F12/DMEM (Biochrom), 10% FCS (Sigma), 60 IU/ml penicillin/streptomycin) or osteogenic media (MO) (MN + 50 μM L-ascorbic acid 2-phosphate, 10 mM β-glycerophosphate, 10 nM dexamethasone) alone or with addition of one of the growth factors from the BMP superfamily (100 ng/ml): rhBMP-2, rhBMP-7 (Biomol, Germany) or rhBMP-9 (Bio-Rad, Germany). The media were changed every second day. Microscopic observations were performed using Axiovert 40 CFL equipped with AxioCam ERc 5 s (Carl Zeiss, Germany) after one day and after 1, 2 and 4 weeks of culture. Images were taken using AxioVision SE64 Rel. 4.9 software (Carl Zeiss, Germany). 6 cultures per one experimental group and time point were prepared and evaluated from each patient (90 cultures per patient).

### Cell viability

Cell viability of the 3D fat cultures was determined using a water-soluble tetrasolium-1 (WST-1) reagent (Roche, Germany). The assay was performed after 24 hours and subsequently after 1, 2 and 4 weeks of culture. Briefly, the culture medium was discarded. The fresh growth medium was mixed with WST-1 at 10:1 (v/v), added to the wells with 3D cultures or to the empty wells (blanks) and incubated for 2 hours at 37 °C in 5% CO_2_. After the incubation, 100 μl of medium-WST-1 mixture was transferred to a 96-well culture plate and the absorbance was read at 450 nm using Synergy HT microplate reader and Gen 5 2.03 software (BioTek, Germany). The assay was repeated 7 times in the independent cultures.

### Histology

Fresh fat tissue samples or 3D fat cultures were embedded in Tissue-Tek O.C.T.^TM^ Compound (Sakura, Germany) and frozen immediately in liquid nitrogen. Tissue sections of 14 μm were cut by the cryomicrotome (CM 3050, Leica, Germany), transferred onto Superfrost^TM^ Plus microscope slides (Thermo Scientific, USA) and air-dried. All samples were prepared in triplets.

#### Alizarin red S staining

Deposition of calcium in the tissue was evaluated by means of Alizarin red S staining. The sections were fixed with 4% Paraformaldehyde (PFA) for 15 minutes at room temperature (RT) and rinsed with distilled water. They were then stained with freshly prepared 40 mM alizarin red S (Sigma) solution, pH 4,1 for 10 minutes at RT and washed in PBS. The samples were dehydrated in 100% Ethanol, cleared in Rothistol (Roth, Germany) and mounted with DPX (Merck).

#### Von Kossa staining

Slides fixed with PFA 4% and washed in distilled water were stained in 5% silver nitrate solution (Sigma) in the darkness for 5 minutes. They were then washed with distilled water and treated with 1% pyrogallic acid (Sigma) for 5 minutes. After washing and fixing with 5% NaOH for 4 minutes, the samples were stained with May-Grünwald stain (Sigma) for 10 minutes, dehydrated with 100% Ethanol, cleared with Rothistol and mounted with DPX.

### RNA isolation and quantitative real-time PCR

The expression of ALP and OCN were analysed in combination with the glyceraldehyde-3-phosphate dehydrogenase (GAPDH) after 1, 2 and 4 weeks of culture. Total mRNA was extracted using QIAzol Lysis Reagent (Qiagen, Gemany) according to manufacturer’s protocol. Briefly, the cultures were frozen in liquid N_2_ and homogenised using the Mikro-Dismembrator S (Sartorius Stedim Biotech, Germany). Subsequently, 1 ml QIAzol Lysis Reagent was added to the homogenate, resuspended and mixed with chloroform. After centrifugation, the upper aqueous phase was transferred to a new tube and mixed with isopropanol. The mRNA containing pellet was washed twice with 75% Ethanol, air-dried and redissolved in RNase-free water. After the incubation at 37 °C for 20 minutes RNA concentration and purity were assessed with the spectrophotometer (NanoDrop^TM^ Lite, Thermo Scientific).

cDNA synthesis was performed for 15 minutes at 42 °C using Thermocycler (T100, Bio-Rad, Germany) in a reaction mixture containing 300 ng total RNA, buffers, Quantiscript reverse transcriptase and RT primer mix from QuantiTect Reverse Transcription-Kit (Qiagen, Germany) adjusted with RNase-free water to a total volume of 20 μl.

Amplification of the cDNA products was performed using the FastStart Essential DNA Green Master (Roche Applied Science, Germany) and the Light Cycler 96 (Roche, Germany). The reaction mixture contained 2,5 μl cDNA, 300 ng primer, 5 μl FastStart Essential DNA Green Master and RNase free water to a final volume of 10 μl. Primer sequences: GAPDH forward TGCACCACCAACTGCTTAGC, reverse GGCATGGACTGTGGTCATGAG (annealing temperature 60 °C); ALP forward TCAAGGGTCAGCTCCACCACA, reverse ATTGGCCTTCACCCCACACA (60 °C); OCN forward CCCAGGCGCTACCTGTATCAA, reverse CTGGAGAGGAGCAGAACTGG (64 °C). Primers were obtained from Metabion (Germany). Amplification was performed in triplicates in 96 well plates (Roche, Germany). Relative gene expression between samples was calculated using the 2^−ΔΔCt^ method, considering GAPDH as a housekeeping gene and untreated fat tissue as a calibrator.

### Immunofluorescent staining

The tissue sections were fixed with 4% PFA and washed in PBS with 0,1% Brij (Sigma). Non-specific binding sites were blocked by 5% bovine serum albumin (BSA) for 30 minutes. Thereafter, primary antibody was added and incubated overnight. All primary antibodies (OCN, OPN, Scl) were mouse monoclonal IgGs from R&D Systems (Germany) used in concentrations: 25 μg/ml (OCN, OPN) and 10 μg/ml Scl. For negative controls, the first antibody was omitted. After washing off unbound antibody and permeabilization with 0,1% Triton X-100 solution (Sigma) in PBS the secondary antibody goat antimouse IgG conjugated with Alexa Fluor 488 (Invitrogen, Germany) in the dilution 1:400 was added for 30 minutes, then intensively washed. Finally, the nuclei of cells were stained with Hoechst 33342 (Life Technologies, Germany). The slides were mounted with Fluoromount W (Serva Electrophoresis, Germany), air-dried and stored in darkness at 4 °C.

Microscopy was performed with the Zeiss Axioskop 40 equipped with appropriate filter sets and AxioCam MRc 5. Images were obtained with an Axio Vision, Rel. 4.9 software (Carl Zeiss, Germany).

### Statistical data analysis

Data analysis was performed with Microsoft Excel 2010 and Prism 5.02 software for Windows. Values were presented as mean 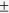 standard error. The Mann-Whitney U-test was used to determine differences between compared groups as a Gaussian distribution could not be assumed. The level of significance was indicated by * for p < 0.05, ** for p < 0.01 and *** for p < 0.001.

## Additional Information

**How to cite this article**: Bondarava, M. *et al*. Osseous differentiation of human fat tissue grafts: From tissue engineering to tissue differentiation. *Sci. Rep.* **7**, 39712; doi: 10.1038/srep39712 (2017).

**Publisher's note:** Springer Nature remains neutral with regard to jurisdictional claims in published maps and institutional affiliations.
